# Oblique Triangular Flap Coverage for Fingertip Amputations: A Malaysian Outcome Case Series

**DOI:** 10.7759/cureus.79210

**Published:** 2025-02-18

**Authors:** Carolyn Choong Yoke Lin

**Affiliations:** 1 Orthopedics Department, Hospital Sultanah Bahiyah, Alor Setar, MYS

**Keywords:** digital amputation, fingertip, fingertip amputations, fingertip injury, fingertip trauma, hand injury, malaysia, pulp loss, triangular oblique flap, venkataswami flap

## Abstract

Fingertip amputations are complex injuries that may involve the loss of soft tissue, pulp, nailbed, tendon, and bone. Many surgical techniques have been described over the years to address these fingertip defects. The choice and method of defect coverage depends on the amount of soft tissue and bone loss. We present a case series of eight fingertip amputations that were treated with oblique triangular flap coverage. Our patients had a minimum of two months follow-up and were assessed at one year postoperatively. The favorable results observed in this series have reinforced our confidence in the reliability of this treatment technique for fingertip amputations in our Malaysian population.

## Introduction

Anatomically, the fingertip is the portion of the terminal digit located distal to the insertions of the extensor and flexor tendons [1]. It plays a crucial role in fine motor function, pinch, grip, and tactile sensation, all of which are essential for performing daily tasks. Injuries to the fingertip not only impair a patient’s ability to perform daily activities but may also negatively impact their quality of life. Thus, achieving optimal reconstruction is vital for restoring both form and function.

Fingertip injuries can result from various trauma mechanisms, including crush, laceration, and avulsion injuries. These injuries often involve the loss of soft tissue, pulp, nailbed, tendon, and bone, leading to defects in the terminal digits. Treatment options for these defects vary based on the degree of tissue loss and the size of the defect and can range from nonoperative to operative approaches [1].

Over the years, numerous surgical flap techniques have been developed and described for fingertip reconstruction, including the V-Y Atasoy flap, Kutler flap, Moberg flap, and Venkataswami flap [2-7]. The choice of technique depends on factors such as the size and orientation of the defect, the degree of tissue loss, and the patient’s functional requirements. The oblique triangular flap, introduced by Venkataswami and Subramanian in 1980, has gained popularity due to its ability to preserve finger length, provide sensate coverage, and avoid donor site morbidity [2]. This technique is particularly advantageous in cases of oblique amputations, where other flaps may not provide adequate coverage [2,8].

In our case series, we focused on the oblique triangular (Venkataswami) flap, and by evaluating outcomes such as functional recovery, patient satisfaction, and complication rates, we aim to provide further insight into the viability of this technique as a standard treatment option in our Malaysian population.

## Materials and methods

From January to October 2023, a total of eight fingers sustained Allen type II-IV fingertip amputations [9]. All eight fingers were treated operatively and underwent oblique triangular flap closure for their defects. These patients were followed up and assessed one year after surgery. Consent was obtained from all patients involved for the purpose of including their case details in this publication. All patients involved agreed to participate and consented voluntarily.

All cases were operated on under general anesthesia, except for one patient who agreed to a local finger block. We routinely perform this procedure under loupe magnification and with a tourniquet. After routine preparations, a finger tourniquet is applied, and wound debridement of devitalized tissue is performed without any bone shortening. A triangular flap was marked as described by Venkataswami [2].

**Figure 1:**
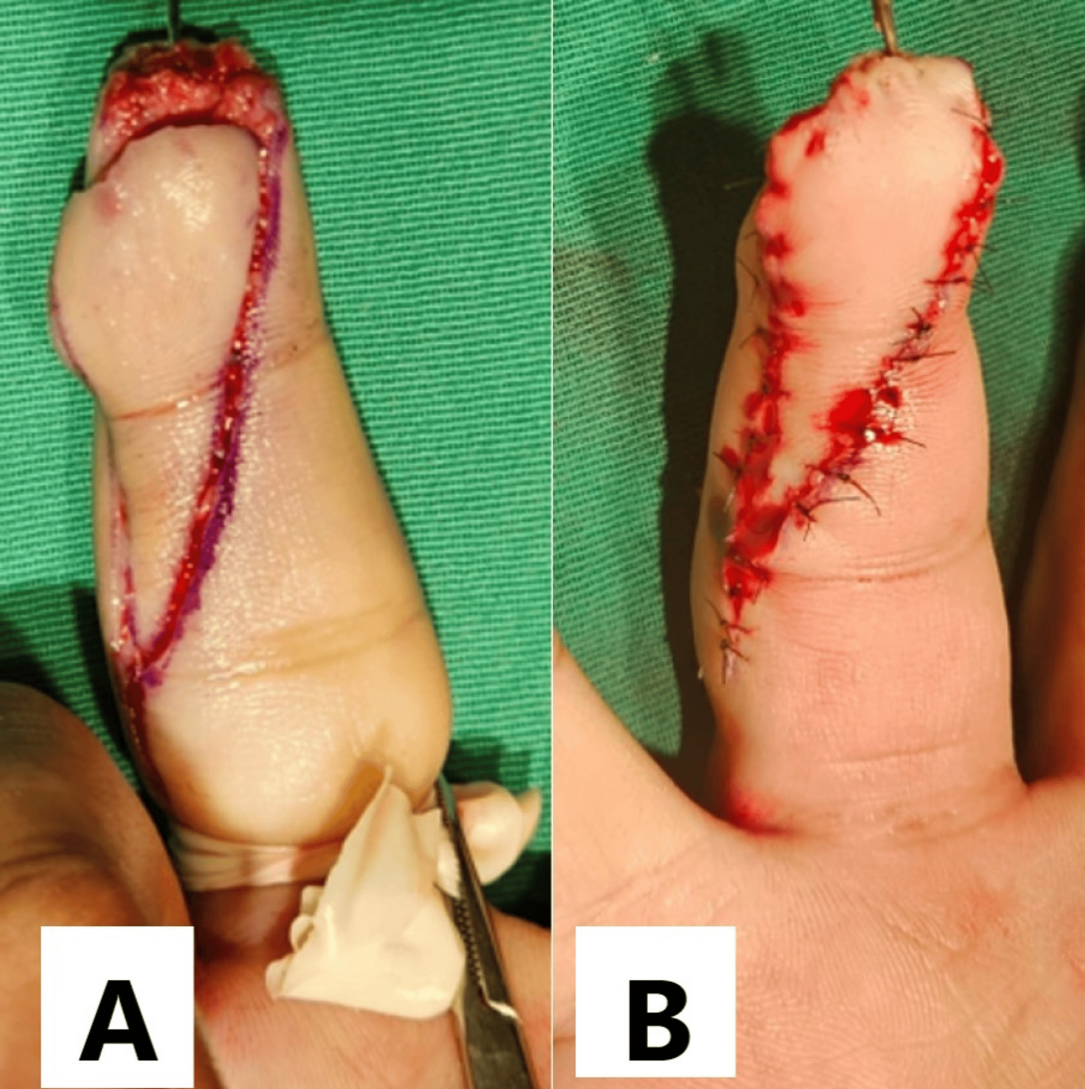
Intraoperative pictures of an oblique triangular flap (A) A finger tourniquet is applied, and the flap is mobilized distally to cover the defect. (B) The oblique triangular flap has been sutured using nonabsorbable sutures

The skin flap length required for mobilization was usually 2 to 2.5 times the length of the base of the wound. The flap was then incised with a size 15 blade, raised, and separated from the underlying fibrous tendon sheath using tenotomy scissors. We took care not to injure the digital neurovascular bundle, which was intended to be incorporated into the skin flap, by performing careful and meticulous dissection. Proximally, the neurovascular bundle was dissected and freed to allow for the advancement of the skin flap distally. After releasing the tourniquet, the circulation to the skin flap was checked and hemostasis was secured. The oblique triangular flap was then advanced over the amputation site for closure. All flaps were secured with nonabsorbable 5/0 sutures (Figure 1).

**Figure 2:**
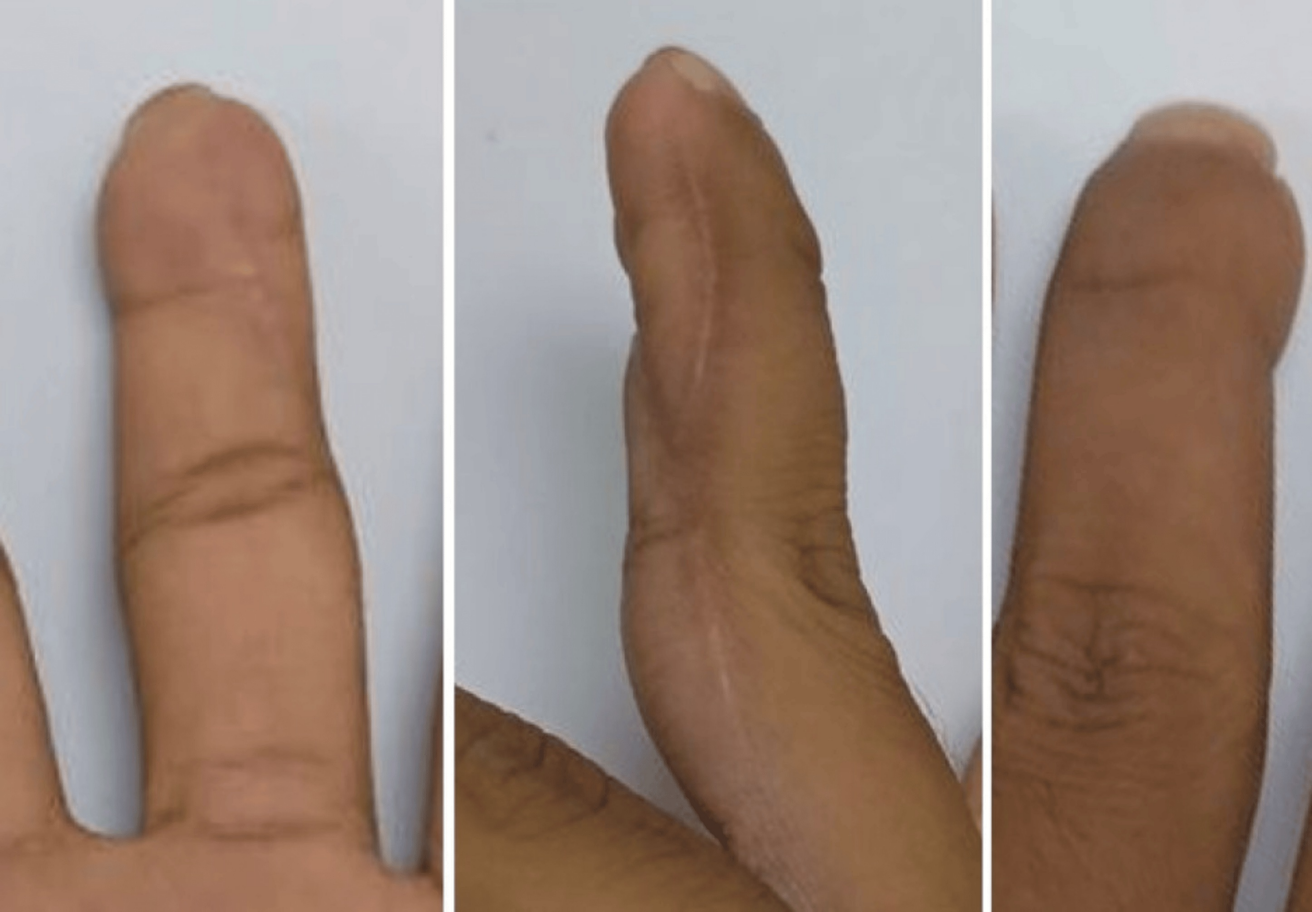
Postoperative images of finger A at one year (volar, lateral, and dorsal views)

Postoperatively, all patients were discharged the next day except for the patient who was operated on under local anesthesia; he was discharged the same day as the surgery. Patients had their first clinic visit at two weeks postoperatively for suture removal and referrals to hand therapists. Subsequent follow-ups were done at four- to six-week intervals. All patients were followed up for a minimum of two months and assessed at one year postoperatively (Figure 2). We measured patients’ outcomes using the Fingertip Injuries Outcome Assessment Score (FIOS) [10].

## Results

From January 2023 to October 2023, a total of eight fingers sustained Allen type II-IV fingertip amputations. The patients, aged between 18 and 50 years, were predominantly male. The most frequently injured fingers were the middle and index fingers. The wound patterns varied, including transverse, radial volar oblique, and ulnar volar oblique types. Most injuries involved the nondominant hand, with the exception of two fingers. The majority of these injuries were due to work-related accidents, while one case resulted from a motor vehicle accident in which the patient lost control of his motorbike, causing his fingertip to be crushed by the handle. Patient outcomes were assessed using the Fingertip Injuries Outcome Score (FIOS) [10], with results summarized in Table 1.

**Table 1:** Outcome results of oblique triangular flap and postoperative complications MF: middle finger; IF: index finger; RF: ring finger; PIP: proximal interphalangeal; LA: local anesthesia; GA: general anesthesia

Finger	Finger involved	Level of amputation	Hand dominance injured	Complications	Wound pattern	Type of anesthesia	Fingertip Injuries Outcome Score (FIOS) value	Fingertip Injuries Outcome Score (FIOS) result
A	Left MF	Allen type III	Nondominant hand	Uneventful	Transverse	GA	12	Excellent
B	Left IF	Allen type II	Nondominant hand	Uneventful	Volar oblique	GA	11	Excellent
C	Left MF	Allen type III	Nondominant hand	PIP joint contracture	Radial volar oblique	GA	15	Good
D	Left RF	Allen type II	Nondominant hand	Uneventful	Radial volar oblique	GA	10	Excellent
E	Left LF	Allen type II	Nondominant hand	Uneventful	Radial volar oblique	GA	10	Excellent
F	Left IF	Allen type II	Nondominant hand	Uneventful	Ulnar volar oblique	GA	11	Excellent
G	Right MF	Allen type II	Dominant hand	Uneventful	Transverse	LA	12	Excellent
H	Right IF	Allen type IV	Dominant hand	Uneventful	Ulnar volar oblique	GA	19	Fair

## Discussion

The optimal treatment for fingertip amputations remains a subject of ongoing debate among hand surgeons due to the various approaches available for managing these defects. The debate often centers on the choice between surgical and nonsurgical options. Nonsurgical management, such as healing by secondary intention with semiocclusive dressings, is often preferred for smaller defects with well-vascularized wound beds [9,11]. In contrast, surgical techniques, including local flaps, skin grafts, and replantation, are considered for larger or more complex injuries requiring tissue coverage and structural support [2-5]. There are also studies that have explored the role of bioengineered tissue substitutes and composite grafts in fingertip reconstruction as alternatives to traditional flaps [12-13]. Nevertheless, universally, the majority of studies have emphasized the importance of patient-specific factors such as age, occupation, and mechanism of injury in determining the most appropriate treatment strategy [1,10,12].

In 1980, Venkataswami and Subramanian introduced the oblique triangular flap for the reconstruction of fingertip amputations, particularly those with obliquely placed wounds [2]. This method has gained widespread use due to its favorable and reproducible outcomes. The oblique triangular flap provides a stable, sensate, and cosmetically acceptable fingertip, while also avoiding donor site morbidity, a significant advantage in populations like Malaysia.

One of the key benefits of the oblique triangular flap is its ability to preserve finger length without requiring bone shortening. The preservation of finger length will provide mechanical advantage and fine motor skills required for tasks like grasping, manipulating tools, or performing repetitive motions. This would in turn improve grip strength and dexterity which are vital for patients engaged in manual labor [14]. The Venkataswami flap can achieve this length preservation because the flap can be advanced distally to adequately cover the wound without tension, which is crucial for optimal healing and function [2].

In contrast to the V-Y plasty technique described by Atasoy, which may offer limited flexibility in wound coverage, the triangular oblique flap allows for the mobilization of the neurovascular bundle [3]. The nerves and blood vessels, critical for skin survival and sensation, are incorporated into the flap itself. This incorporation ensures that the neurovascular bundle remains intact and viable, contributing to the successful integration of the flap [2,15].

As a result, the triangular oblique flap enables greater flap advancement and provides coverage for larger wounds. This approach not only enhances cosmetic and functional outcomes but also ensures the survival of the skin flap by maintaining a robust blood supply and innervation. Overall, this technique offers a more effective solution for managing fingertip amputations, providing both coverage and preservation of bone length.

In our case series, we measured patient outcomes using the Fingertip Injury Outcome Score (FIOS), introduced by Jerome and associates in 2022 [10]. This scoring system was designed to evaluate the outcomes of fingertip injuries posttreatment by assessing overall function and recovery. The FIOS incorporates both objective and subjective assessments, considering a total of 10 factors: nail, finger length, pulp, bone, sensation, range of motion, and grip strength as objective assessments; and pain, cosmesis, and return to work as subjective assessments.

The FIOS scores various aspects of fingertip function and appearance on a scale, with the total score reflecting the overall success of the treatment. Scores of 12 and below indicate excellent outcomes, while scores of 24 and above suggest poor outcomes with residual issues such as pain, numbness or hyperalgesia, joint stiffness, poor grip strength, and inability to return to work. This scoring system is valuable for clinicians and researchers to objectively measure the effectiveness of different treatment methods for fingertip injuries and to compare outcomes. It is straightforward and easy to apply in a busy clinic setting [2,16].

Moving forward, we would like to incorporate patient-reported outcome measures (PROMs) into the FIOS to further enhance its utility. There have been modifications to the FIOS to include the addition of PROMs, which provide further insight into the patient’s perspective on their recovery and satisfaction with treatment. By including PROMs, the modified FIOS offers a more comprehensive evaluation of both functional and aesthetic outcomes, enhancing its utility in clinical practice for assessing and comparing different treatment modalities [16-17].

In our case series, the majority of flap outcomes were favorable at one year, with FIOS results classified as "excellent" for six of the eight fingers. This reflects the reliability of the Venkataswami flap in providing a stable, sensate, and cosmetically acceptable fingertip. Our results also demonstrate that functionally, these patients with the Venkataswami flap were able to regain their ability to return to work with good grip strength and range of motion in the injured finger.

Nevertheless, there was one finger (Finger C) with a FIOS score of 15 (good). This was due to the patient developing a contracture of the proximal interphalangeal (PIP) joint in injured finger. Despite his best efforts in doing hand therapy, the PIP joint contracture did not improve. The patient was subsequently offered the option of joint contracture release. He declined further surgery but was still able to return to his regular work.

We had another finger (Finger H) that scored 19 (fair). This was attributed to the fact that the patient had developed stiffness in the distal interphalangeal (DIP) joint of the injured finger. This patient also had the worst presenting fingertip injury (Allen type IV) in our case series. The absence of a nail plate, and shortened bone length due to the initial level of amputation (Allen type IV) had increased the scoring of Finger H, despite him being able to return to his regular work postoperatively.

Overall, our case series demonstrated good to excellent outcomes with the use of the oblique triangular flap. This flap proved to provide a stable, cosmetically acceptable, painless, and sensate fingertip. The procedure was completed in under one hour under local anesthesia, particularly for single-digit injuries, as exemplified by Finger G (Table 1).

For patients undergoing local anesthesia, this approach allows for same-day discharge, thereby reducing the need for prolonged hospital admissions and associated costs. Additionally, the low complication rates associated with this flap contribute to shorter hospital stays and further cost savings [18-19]. In an ideal hospital setting where operating theaters are readily available for short emergency procedures for fingertip injuries, we believe this technique could significantly improve hospital bed occupancy by reducing admissions and minimizing hospital stays. Further comprehensive studies are needed to assess the potential cost-effectiveness of this procedure, taking into account the reduced complication rates and associated savings on hospital expenditures.

## Conclusions

The oblique triangular flap remains a reliable and reproducible technique for reconstructing fingertip amputations, offering significant advantages, such as no donor site morbidity and the potential for an early return to work. Unlike conservative dressings, which often take longer to heal and result in a delayed return to work, this surgical approach provides faster recovery, allowing patients to resume their daily activities sooner. While new flap techniques are exciting, sometimes the tried-and-true methods prove to be more valuable, as demonstrated by our case series. This approach, with its proven stability and reliability, is particularly well-suited for our Malaysian population, providing a superior alternative to conservative dressings.

## References

[1] Kawaiah A, Thakur M, Garg S, Kawasmi SH, Hassan A (2020). Fingertip injuries and amputations: a review of the literature. Cureus.

[2] Venkataswami R, Subramanian N (1980). Oblique triangular flap: a new method of repair for oblique amputations of the fingertip and thumb. Plast Reconstr Surg.

[3] Atasoy E, Ioakimidis E, Kasdan ML, Kutz JE, Kleinert HE (1970). Reconstruction of the amputated finger tip with a triangular volar flap: a new surgical procedure. J Bone Joint Surg Am.

[4] Scerri GV, Park AJ, Hurren JS (1995). A flap for segmental loss of a digital nerve. The Venkataswami flap revisited. J Hand Surg Br.

[5] Adani R, Busa R, Castagnetti C, Bathia A, Caroli A (1997). Homodigital neurovascular island flaps with "direct flow" vascularization. Ann Plast Surg.

[6] Kutler W (1947). A new method for finger tip amputation. J Am Med Assoc.

[7] Moberg E (1964). Aspects of sensation in reconstructive surgery of the upper extremity. J Bone Joint Surg Am.

[8] Yassin AM, Dash S, Nikkhah D (2022). Workhorse flaps for thumb reconstruction. Plast Aesthet Res.

[9] Allen MJ (1980). Conservative management of finger tip injuries in adults. Hand.

[10] Jerome JT, Malshikare VA (2022). Fingertip injuries outcome score. Plast Reconstr Surg Glob Open.

[11] Krauss EM, Lalonde DH (2014). Secondary healing of fingertip amputations: a review. Hand (N Y).

[12] Sebastin SJ, Chung KC (2011). A systematic review of the outcomes of replantation of distal digital amputation. Plast Reconstr Surg.

[13] Hirase Y (1997). Salvage of fingertip amputated at nail level: new surgical principles and treatments. Ann Plast Surg.

[14] Bardo A, Town K, Kivell TL (2022). The precision of the human hand: variability in pinch strength and manual dexterity. Symmetry.

[15] Evans DM, Martin DL (1988). Step-advancement island flap for fingertip reconstruction. Br J Plast Surg.

[16] M S, Rajput D (2024). A comprehensive study of fingertip injuries at a tertiary care centre-fingertip injury outcome score subset pilot. Int J Res Orthop.

[17] Rob E, Druel T, Jalaguier T, Walch A, Gazarian A (2023). Long-term patient-reported outcome measures of fingertip coverage with a homodigital unipedicle neurovascular island flap. J Hand Surg Eur Vol.

[18] Graff V, Gabutti L, Treglia G (2023). Perioperative costs of local or regional anesthesia versus general anesthesia in the outpatient setting: a systematic review of recent literature. Braz J Anesthesiol.

[19] Öztürk İA, Öztürk K, Orman O, Baydar M, Aykut S, Köse A (2018). Comparison of the cost and efficacy of axillary anesthesia and wide-awake anesthesia in finger surgeries. Med Bull Sisli Etfal Hosp.

